# Small extracellular vesicles (sEVs)-based gene delivery platform for cell-specific CRISPR/Cas9 genome editing

**DOI:** 10.7150/thno.92133

**Published:** 2024-04-28

**Authors:** Sunil Dubey, Zhe Chen, Yuxiao Jarvan Jiang, Austin Talis, Andrei Molotkov, Alessandra Ali, Akiva Mintz, Fatemeh Momen-Heravi

**Affiliations:** 1 Cancer Biology and Immunology Laboratory, Columbia University Irving Medical Center, NY, New York, USA.; 2 Section of Oral, Diagnostic, and Rehabilitation Sciences, Columbia University College of Dental Medicine, Columbia University, New York, NY, USA.; 3 Department of Radiology, Columbia University Irving Medical Center, New York, NY, USA.; 4 Herbert Irving Comprehensive Cancer Center, Columbia University Irving Medical Center, New York, NY, USA.

**Keywords:** CRISPR, Cas, Delivery, sEVs, Genome Editing

## Abstract

Small extracellular vesicles (sEVs) are naturally occurring vesicles that have the potential to be manipulated to become promising drug delivery vehicles for on-demand *in vitro* and *in vivo* gene editing. Here, we developed the modular safeEXO platform, a prototype sEV delivery vehicle that is mostly devoid of endogenous RNA and can efficaciously deliver RNA and ribonucleoprotein (RNP) complexes to their intended intracellular targets manifested by downstream biologic activity. We also successfully engineered producer cells to produce safeEXO vehicles that contain endogenous Cas9 (safeEXO-CAS) to effectively deliver efficient ribonucleoprotein (RNP)-mediated CRISPR genome editing machinery to organs or diseased cells *in vitro* and *in vivo*. We confirmed that safeEXO-CAS sEVs could co-deliver ssDNA, sgRNA and siRNA, and efficaciously mediate gene insertion in a dose-dependent manner. We demonstrated the potential to target safeEXO-CAS sEVs by engineering sEVs to express a tissue-specific moiety, integrin alpha-6 (safeEXO-CAS-ITGA6), which increased their uptake to lung epithelial cells *in vitro* and *in vivo*. We tested the ability of safeEXO-CAS-ITGA6 loaded with EMX1 sgRNAs to induce lung-targeted editing in mice, which demonstrated significant gene editing in the lungs with no signs of morbidity or detectable changes in immune cell populations. Our results demonstrate that our modular safeEXO platform represents a targetable, safe, and efficacious vehicle to deliver nucleic acid-based therapeutics that successfully reach their intracellular targets. Furthermore, safeEXO producer cells can be genetically manipulated to produce safeEXO vehicles containing CRISPR machinery for more efficient RNP-mediated genome editing. This platform has the potential to improve current therapies and increase the landscape of treatment for various human diseases using RNAi and CRISPR approaches.

## Introduction

Small extracellular vesicles (sEVs) are nanosized (70-200 nm) membrane-bound vesicles found in biofluids and secreted by nearly all types of cells in the cellular microenvironment 1-3. They naturally carry biomacromolecules—including different RNAs (mRNAs, regulatory microRNAs (miRNAs)), DNAs, lipids, and proteins—and can efficiently deliver their cargos to recipient cells, mediating cellular communication and functionality 4. Previous work by our group and others has shown the advantages of using sEVs for drug delivery. Small extracellular vesicles 1) are small and have a high efficiency for delivery due to their similarity to cell membranes; 2) are biocompatible, non-immunogenic, and non-toxic, even with repeated *in vivo* injections 5; 3) are stable even after several freeze and thaw cycles; 4) contain a lipid bilayer that protects their protein and RNA cargos from enzymes such as proteases and RNases 2; 5) have a slightly negative zeta potential, leading to long circulation 6; and 6) exhibit an increased capacity to escape degradation or clearance by the immune system 7, 8. Thus, exogenous sEVs are being developed for their potential to deliver RNA interference (RNAi), miRNAs, and mRNA by our group and others 5, 9, 10, and hold great potential for future therapeutic uses.

The CRISPR/Cas genome editing system has been utilized extensively in recent years for its ability to produce targeted genome editing. The technology is rapidly maturing as a clinical grade technology for select genetic diseases and is actively being explored for a myriad of diseases. However, challenges remain in its widespread therapeutic use, partly due to the lack of a targeted, reliable, and safe delivery method for the requisite gene editing components. sEVs present a promising delivery vehicle for genome editing cargo, but innovative approaches are needed to overcome several challenges in order to successfully utilize them as a scalable, targeted, and reliable vehicle for CRISPR/Cas machinery to efficiently facilitate gene editing without unwanted biological effects or uncharacterized endogenous cargo.

CRISPR-based editing induces double-stranded DNA (dsDNA) breaks in a targeted fashion, prompting resultant DNA repair via either nonhomologous end joining (NHEJ) or homology directed repair (HDR). NHEJ occurs without a template and thus may be exploited for gene knockout by deletion, frameshift or the introduction of new STOP-codons via mutation 11, 12. NHEJ requires a minimal Cas endonuclease and single guide RNA (sgRNA). Cas plus a single sgRNA is adequate for gene disruption/mutation, while Cas plus two sgRNAs are needed for gene deletion. HDR, which inserts new genetic material, requires Cas, sgRNAs along with a 'spare' DNA template for targeted genetic insertion. HDR thus provides an opportunity to knock-in specific DNA sequences at targeted locations in the genome to correct genetic diseases or introduce new functional proteins, although it is much less efficient compared to NHEJ 13, 14.

One of the main challenges of using CRISPR/Cas for the treatment of human diseases is the lack of efficient delivery methods for the requisite complex multicomponent machinery that can target specific cells and organs *in vivo* and avoid gene edits in non-target cells or genetic loci 15. CRISPR/Cas can be delivered locally or systemically via gene-based delivery (DNA plasmids or viruses encoding Cas and sgRNAs), RNA-based delivery (mRNA encoding Cas along with a synthetic sgRNA), or formed Ribonucleoprotein (RNP) complexes consisting of Cas protein prebound to synthetic sgRNA. Of these three methods, RNP-based delivery appears to be superior and most specific to editing the targeted genome site 16 because it does not rely on uncontrolled CAS integration or expression, although it is also the most difficult to implement as a therapeutic due to the complexities of manufacturing and efficiently delivering intact, functional recombinant Cas endonuclease proteins together with the requisite sgRNA and DNA templates specifically to the site of disease.

Although both viral and non-viral approaches have been adopted for *in vivo* delivery of CRISPR/Cas machinery, the effective *in vivo* delivery of multiple CRISPR components into host cells remains a major challenge. Adenovirus (AV) is an efficient transducing agent used for CRISPR/Cas-mediated genome editing, however, this method can elicit a significant immune response in the host 17. Lentiviral vectors are widely used for therapeutic delivery, though their integration into the genome makes them suboptimal for gene editing purposes as long-lasting expression of Cas protein and sgRNA is considered to be unfavorable for the on-target/off-target ratio of indel formation 18. Further, viral vectors are limited in terms of cargo-carrying capacity and cell tropism. These shortcomings present difficulties regarding the distribution and dosage of genome editing nucleases *in vivo*, leading to off-target mutation profiles that may be difficult to predict 18, 19. Non-viral synthetic vectors are another class of delivery vehicles that lack tissue tropism, yet they may provide targeted cell/organ-specific delivery if complexed with targeting moieties such as peptides or antibodies 20. Precise targeting, however, is particularly difficult to achieve, as the incorporation of additional biomolecules to a delivery vector alongside the CRISPR components increases packaging complexity 21. Disadvantages of synthetic delivery vectors include issues with biocompatibility and toxicity, immunogenic potential, and problems with therapeutic cargo release 19. Thus, current implementations of CRISPR/Cas machinery via viral- and non-viral delivery present challenges that prevent the full therapeutic exploitation of gene editing, and novel solutions are needed to translate the scientific progress in gene editing to benefit patients.

Although CRISPR/Cas9 system can be packaged into sEVs by physical methods including electroporation, these approaches could be inefficient, so genetically engineered sEVs have been devised as novel packaging strategies to facilitate cargo loading of sEVs-mediated CRISPR/Cas9 deliver 22-26. Ye et al., demonstrated that efficient packaging was achieved *in vitro* by the fusion between GFP and GFP nanobody in GFP-expressing sEV protein CD63 and GFP nanobody-expressing Cas9 protein 27. Li et al reported that a novel strategy, where sEV surface protein CD9 was genetically fused with Human antigen R (HuR) which recognized the AU rich elements (AREs) expressed by engineered Cas9 mRNA, specifically enriched the encapsulation of Cas9 mRNA into engineered sEVs, demonstrating a promising approach of mRNA cargo loading 28. Additionally, incorporation of inducible heterodimerization partners such as Cryptochrome 2 (CRY2) and Cryptochrome-interacting basic helix-loop-helix 1 (CIB1) or FK506-binding protein (FKBP) and FKBP-rapamycin-binding domain (FRB), expressed on EVs and Cas9 respectively, has been suggested as an approach to cargo loading of Cas9 protein into sEVs 24.

It has been shown that sEVs derived from specific cell types could be used for targeted delivery of CRISPR/Cas9 system 25. MSC-derived sEVs delivered CRISPR/Cas9 system and successfully suppressed oncogenic mutation *KrasG12D in vitro* to reduce tumor growth in pancreatic cancer cells 29. The ovarian tumor-derived sEVs delivered a CRSIPR/Cas9 system editing Poly (ADP-Ribose) Polymerase-1 (PARP-1) selectively to ovarian cancer cells to increase their chemosensitivity and produced synergic cytotoxicity when combined with chemotherapy 30. Dooley et al devised a versatile platform using truncated versions of scaffold proteins, where fusing Cas9 with the N-terminus of BASP1, an EV scaffold protein of MARCKS protein family, directed luminal loading of Cas9 31. These successful results demonstrated the potential of engineering sEVs to enhance targeted delivery, and, though not applied to CRISPR/Cas9, genetically modified sEVs increased the specificity of delivery of siRNA, mRNA, or miRNA 22.

**Figure 1:**
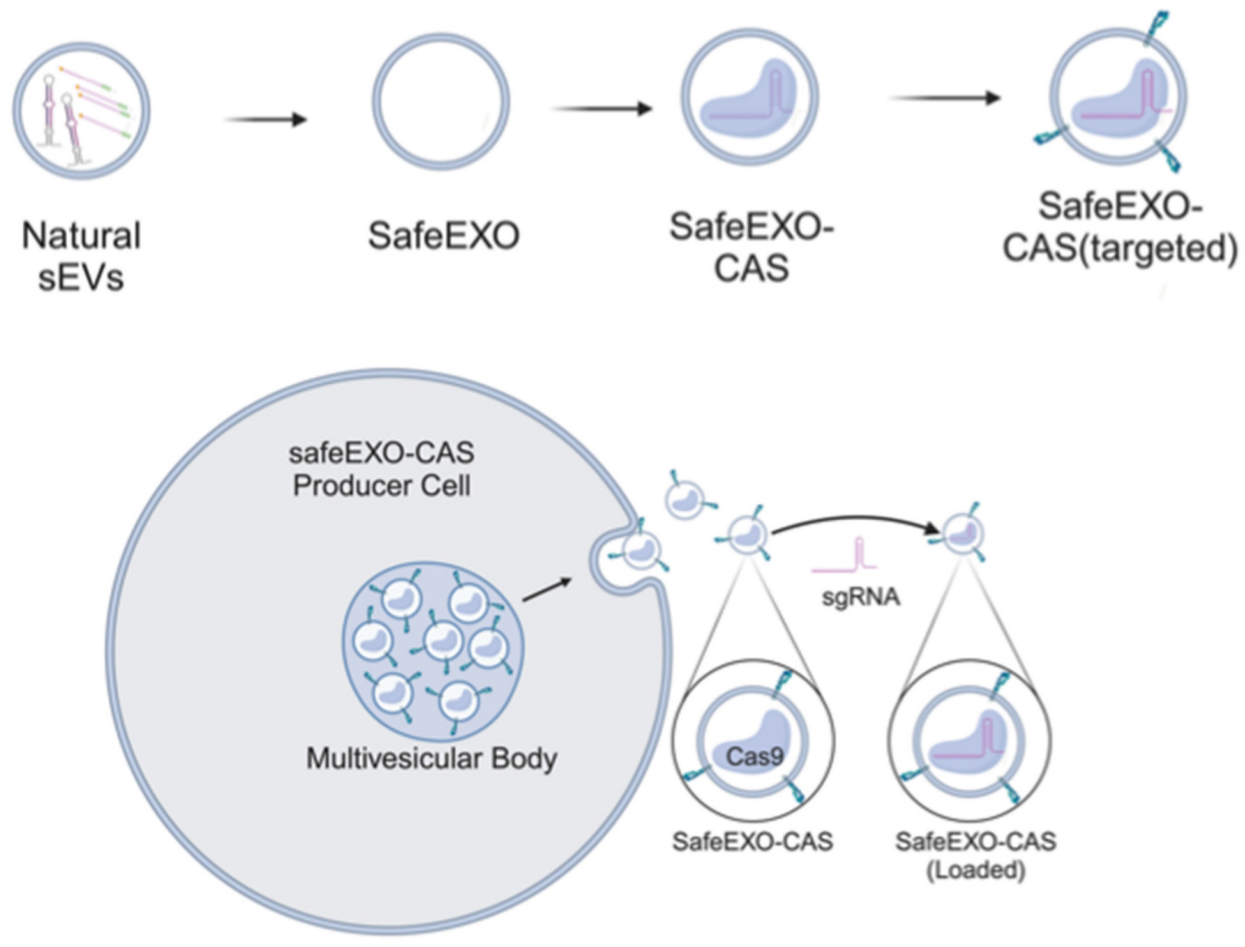
Schematic representation of safeEXO-CAS sEVs.

In this work, we engineered sEVs for effective, targeted, and scalable delivery of CRISPR/Cas machinery and demonstrated efficient gene editing *in vitro* and *in vivo*. We generated a novel sEV-based drug delivery platform **(Figure 1)** engineered (1) to minimize endogenous nucleic acid cargo (safeEXO); (2) to endogenously carry an active Cas9 protein (safeEXO-CAS); and (3) to express tissue specific targeting moieties. Our safeEXO-CAS loaded sEVs efficiently edited recipient cells *in vitro* and *in vivo* using NHEJ-mediated disruption or HDR-mediated insertions without inducing immunogenic response or off-target effects.

## Results

### SafeEXO - Creating sEVs devoid of endogenous RNA

**Figure 2:**
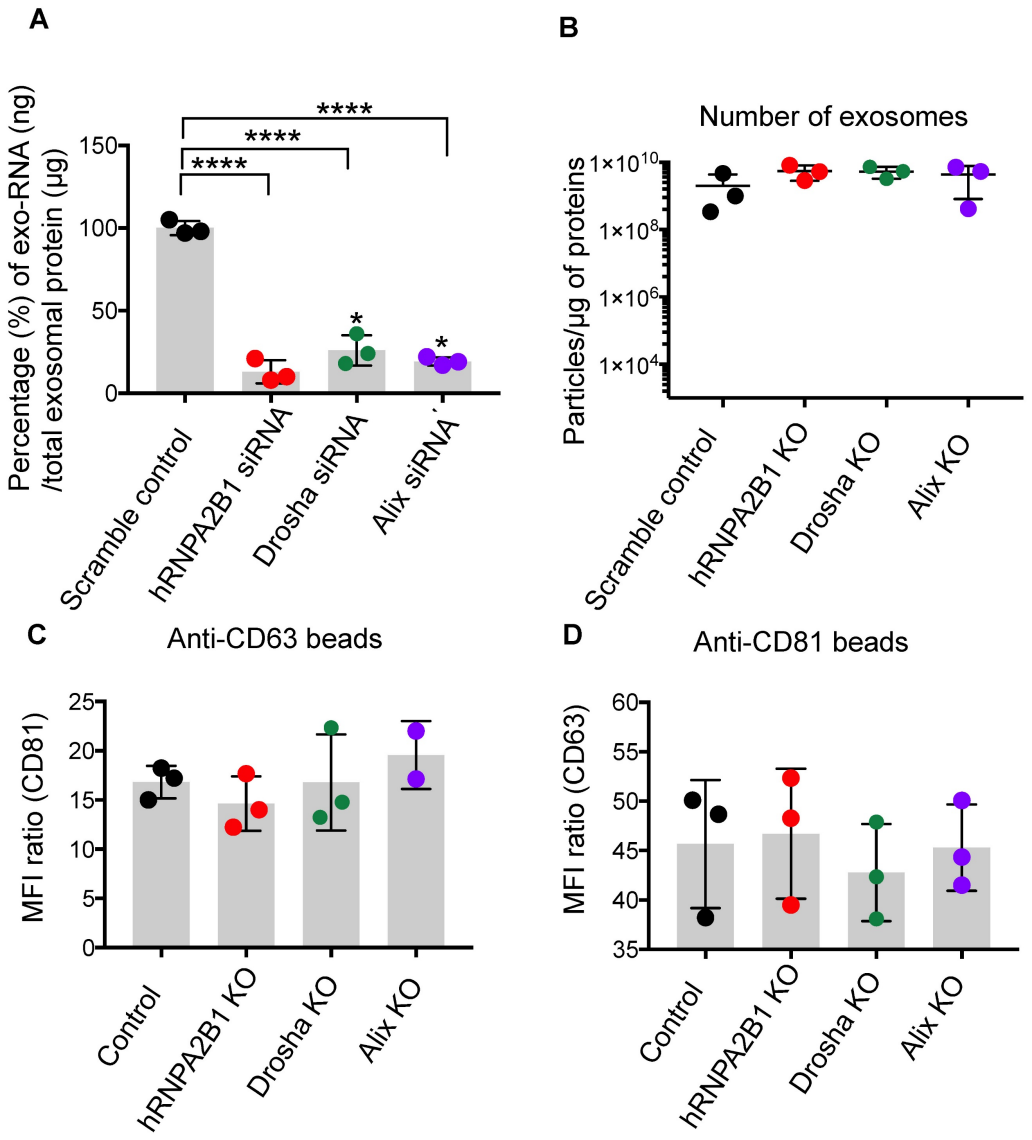
SafeEXO-CAS characterization and preparation.** A** Percentage change of total exo-RNA (ng) normalized based on the protein in sEVs, isolated from siRNA silenced hnRNPA2B1, ALIX, and Drosha THP-1 monocytes. **B** Number of sEVs produced per μg of sEVs protein from hnRNPA2B1, ALIX, and Drosha knockout THP-1 monocytes. **C-D** Flow cytometric analysis of sEVs from hnRNPA2B1, ALIX, and Drosha knockout for the presence of sEV-enriched markers CD63 and CD81. SafeEXO-CAS sEVs were labeled with anti-CD81 or anti-CD63 magnetic beads and the levels of CD81 and CD63 were quantified by flow cytometry.

It is well documented that sEVs, based on their cellular origin, carry specific and considerable amounts of nucleic acids such as mRNA and miRs 32-34. RNA carried by sEVs has been implicated in propagating many diseases, including cancer and infectious diseases. Thus, the presence of significant endogenous biologically active RNA cargo introduces complications in the manufacturing and characterization of sEVs and might introduce potential adverse effects that complicate their use as therapeutic vehicles. Here, we aimed to overcome this limitation by producing sEVs which are almost devoid of endogenous nucleic acid cargo that can be used for the loading and delivery of only the intended miRs, mRNA, and sgRNA being used for therapeutic purposes. To accomplish this in a scalable manner, we knocked out components of the endogenous sEV RNA loading machinery in sEV producer cells. Heterogeneous nuclear ribonucleoprotein (hnRNPA2B1), binds to RNA through the recognition of specific motifs, controlling its loading into sEVs 35. Drosha plays a role in miR processing and loading into sEVs 36. ALIX plays a role in the loading of sEVs with miRs and mRNA 37. Silencing hnRNPA2B1, ALIX, or Drosha resulted in a significant reduction of endogenous RNA in the sEVs and production of sEVs practically devoid of endogenous nucleic acids **(Figure 2A)**. Elimination of hnRNPA2B1, ALIX, and Drosha in producer cells did not change the number or quality of sEVs produced. sEVs isolated from hnRNPA2B1, ALIX, and DROSHA knock-out (KO) cell lines demonstrated normal protein/particle ratio **(Figure 2A)**, and no significant difference between levels of sEV enriched tetraspanins CD63, and CD81 via flow cytometry **(Figure 2C-D)** compared to parental cell sEVs. While producer cells with ALIX KO showed normal growth patterns, cells with Drosha KO and hnRNPA2B1 KO exhibited less proliferation **(Figure S1)**. Thus, sEVs produced from ALIX-KO cells, referred to herein as safeEXO, that are nearly devoid of potentially unwanted endogenous RNA, were used for further experiments and as the base vehicle for delivery of RNA and genome editing machinery. SafeEXO sEVs production was accomplished in 3 independent cell lines, demonstrating the broad applicability of generating sEVs mostly devoid of RNA, including from standard sEVproducer cells or cancer cells (**Figure S2**). The biological significance of the safeEXO platform was tested by exposing cancer cell-derived normal or safeEXO sEVs to CAL27 cells, which have been shown to increase proliferation when exposed to cancer produced sEVs. safeEXO sEVs generated from CAL27 did not increase proliferation of CAL27 cancer cells, in contrast to the natural sEVs that were produced by the same background cell line (**Figure S3**). To confirm that safeEXO vehicles maintained their ability to deliver exogenously loaded RNA into cells, we exposed cells to safeEXO sEVs loaded with miR-155 and demonstrated robust cell uptake and intracellular targeting manifested by downstream functionality **(Figure S4).**

### SafeEXO vehicles engineered to endogenously express Cas9

**Figure 3:**
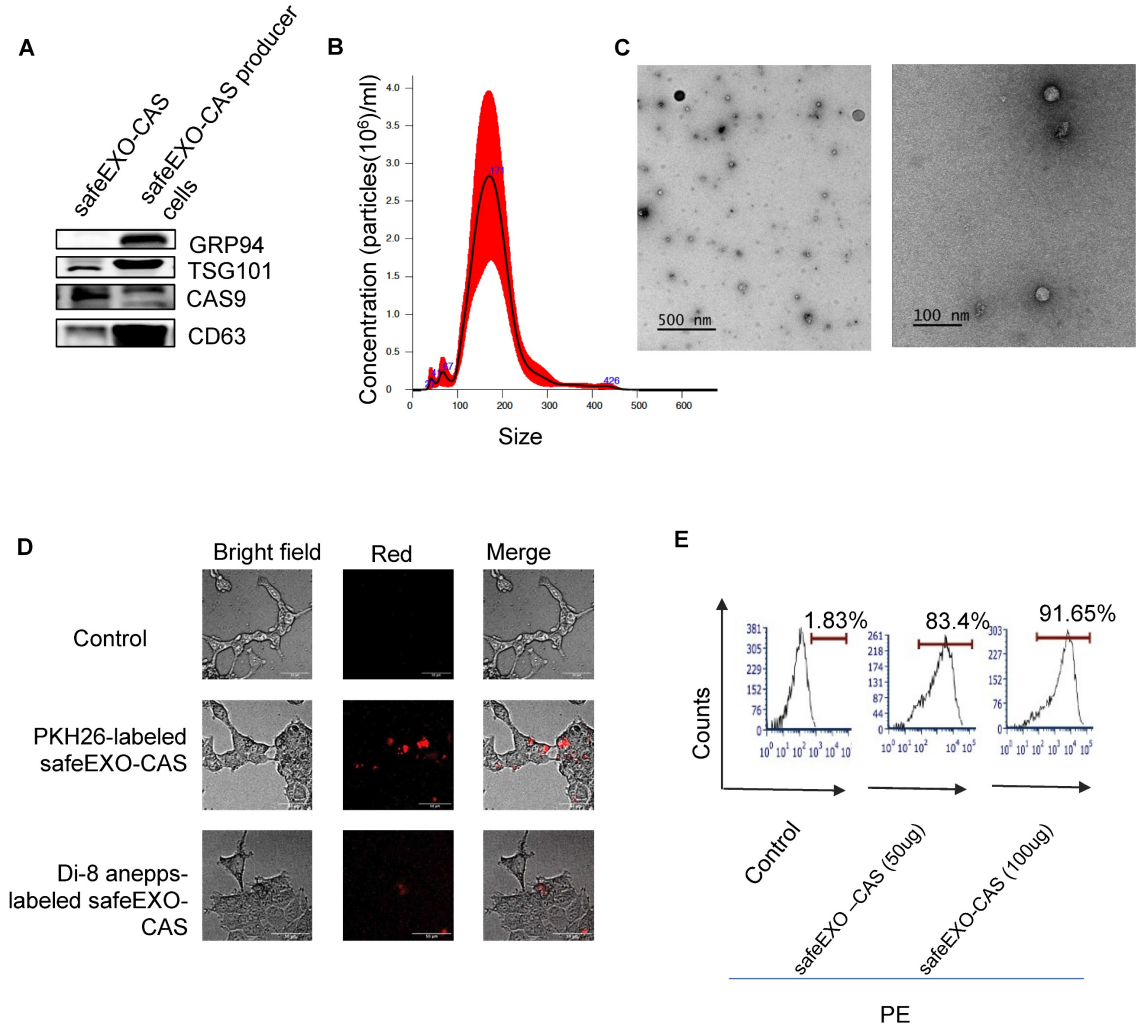
SafeEXO-CAS characterization and uptake. **A** Western blot analysis of sEV markers CD63 and TSG101, along with CAS9 and GRP94 (negative control) in safeEXO-CAS sEVs from THP-1 monocyte producer cells with and without ALIX knockout. **B** Size of safeEXO-CAS sEVs quantified by NanoSight. **C** Transmission electron micrograph of safeEXO-CAS sEVs. **D** Fluorescence microscopy of THP-1 cell line co-cultured with PKH26 and Di-8-ANEPPS labeled safeEXO-CAS after 1 hour. **e** Flow cytometry analysis of Di-8-ANEPPS labeled safeEXO-CAS uptake in THP-1 cell line after 1h co-culture. Experiments were repeated twice and data are represented as mean ± standard deviation (SD). ****p ≤ 0.0001, One-way ANOVA was used for the comparison of multiple groups, and Student's T test was used for pairwise comparison.

Genome editing represents a major interest in basic and translational studies. To generate sEVs with minimal endogenous nucleic acids, we knock out ALIX in Cas9-expressing THP-1 cells to generate RNA-cleared, sEVs containing endogenous Cas9 (safeEXO-CAS). sEVs that express canonical markers (CD63 and TSG101) and CAS9 **(Figure 3A)**. SafeEXO-CAS sEVs showed a mean size of around 170 nm measured by Nanosight analysis and transmission electron microscopy (TEM) **(Figure 3B-C).** To confirm cellular targeting and internalization by target cells, safeEXO-CAS vehicles were labeled with PKH26 or Di-8-ANEPPS. PKH26 constitutively fluoresces but Di-8-ANEPPS conditionally fluoresces upon internalization into the target cell 38, thus allowing the target uptake of fluorescently labeled safeEXO-CAS sEVs to be visualized and quantified via microscopy and flow cytometry. After a 1h co-culture, safeEXO-CAS sEVs were taken up efficiently by the recipient cells **(Figure 3D)**. Flow cytometry confirmed a dose-dependent increase in the safeEXO-CAS uptake after co-culture **(Figure 3E)**.

### SafeEXO-CAS vehicles mediate efficient CRISPR-based NHEJ genomic editing

**Figure 4:**
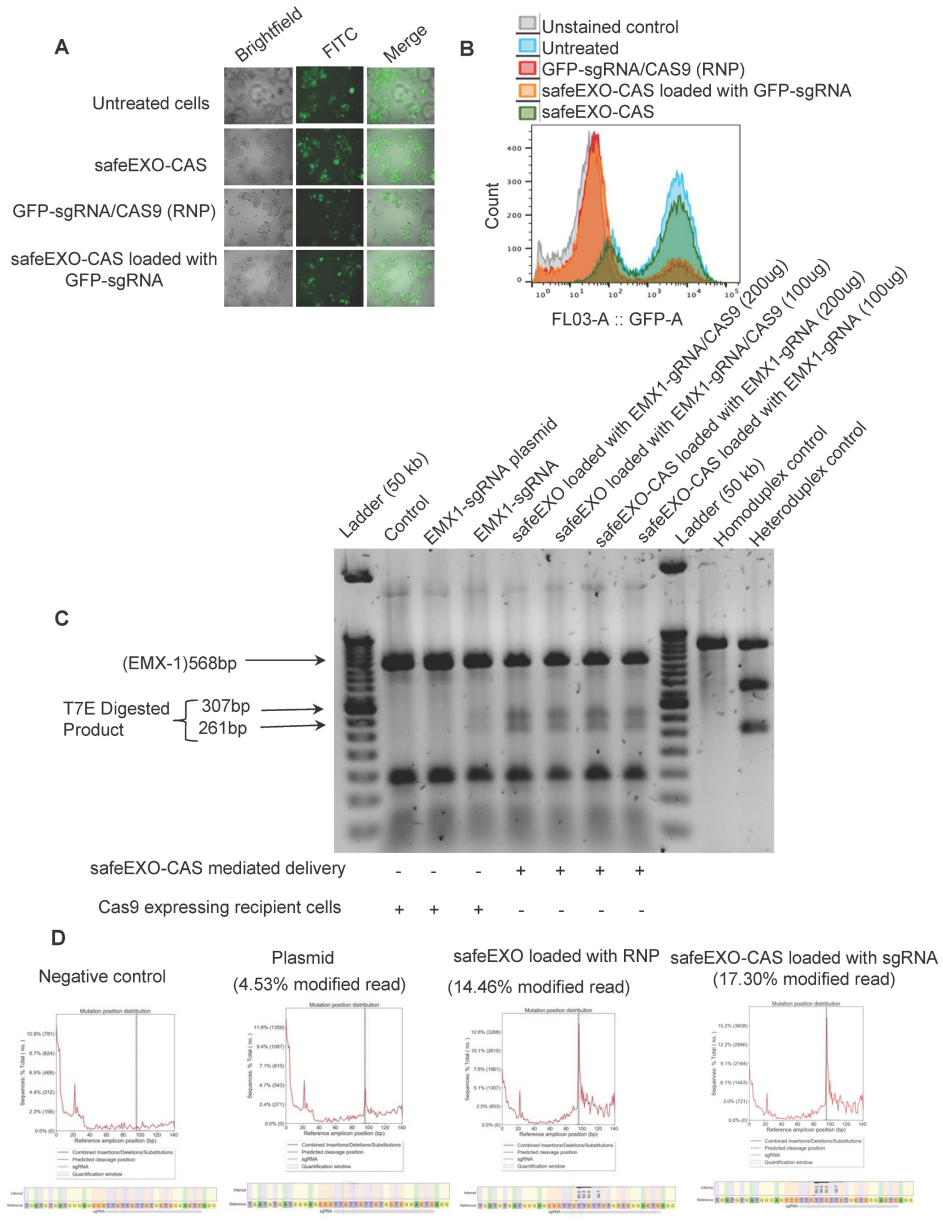
SafeEXO-CAS-mediated non-homologous end joining (NHEJ) genome editing.** A** Fluorescence microscopy and **B** FACS analysis of GFP expressing HEK293T cells treated with safeEXO-CAS, GFP-sgRNA/CAS9 *ribonucleoprotein* (RNP), and safeEXO-CAS loaded with GFP-sgRNA. **C** T7 endonuclease assay against the EMX1 in Cas9 expressing THP-1 cells and in THP-1 cells. Cells were treated with different concentrations of safeEXO with CAS9/EMX1 RNP (200ug and 100ug) or safeEXO-CAS loaded with EMX1-sgRNA (200ug and 100ug). EMX1-sgRNA plasmid and EMX1-sgRNA as an RNP were used as positive control. **D** The percentages of indel induction in EMX1 gene were quantified in negative control, EMX1-sgRNA plasmid, safeEXO loaded with CAS9/EMX1 RNP and safeEXO-CAS loaded with EMX1-sgRNA based on deep sequencing of EMX1 locus.

Next, safeEXO-CAS sEVs were tested for their potential to efficiently induce NHEJ genome editing using a GFP HEK293T reporter cell line that was exposed to safeEXO-CAS sEVs loaded with sgRNA directed at disrupting GFP. Genome editing was demonstrated by a significant decrease in GFP expression in target cells as demonstrated by fluorescent microscopy and flow-cytometry **(Figure 4A-B)**. Indel induction in the target cell line by T7 endonuclease assay **(Figure S5)**.

NHEJ genome editing by safeEXO-CAS sEVs was independently confirmed on a different genetic locus in a second cell line. For these confirmatory studies, we used monocytes in suspension culture that are generally refractory to DNA transfection and other gene delivery methods compared to HEK-293 cells. SafeEXO-CAS sEVs were loaded with sgRNA targeting the EMX1 locus and exposed to targeted monocytes *in vitro*. 72 h after safeEXO-CAS exposure, genome editing at the EMX1 locus was assessed in the bulk cellular population of monocytes using the T7 endonuclease assay. The results demonstrated that safeEXO-CAS sEVs loaded with EXM1-targeted sgRNA efficiently edited the EXM1 locus and were superior to gene therapy using a plasmid encoding CAS9 and EXM1-targeted sgRNA **(Figure 4C)**. To confirm these findings in separate experiments was performed with a subset of conditions and the results were subjects to and deep-sequencing of the EMX1 locus. safeEXO-CAS sEVs loaded with sgRNA and safeEXO vehicles externally loaded with recombinant Cas9 protein and sgRNA were significantly more efficient compared to the plasmid delivery **(Figure 4D)**.

### SafeEXO-CAS vehicles with complex payloads mediate efficient CRISPR-based HDR genomic editing

**Figure 5:**
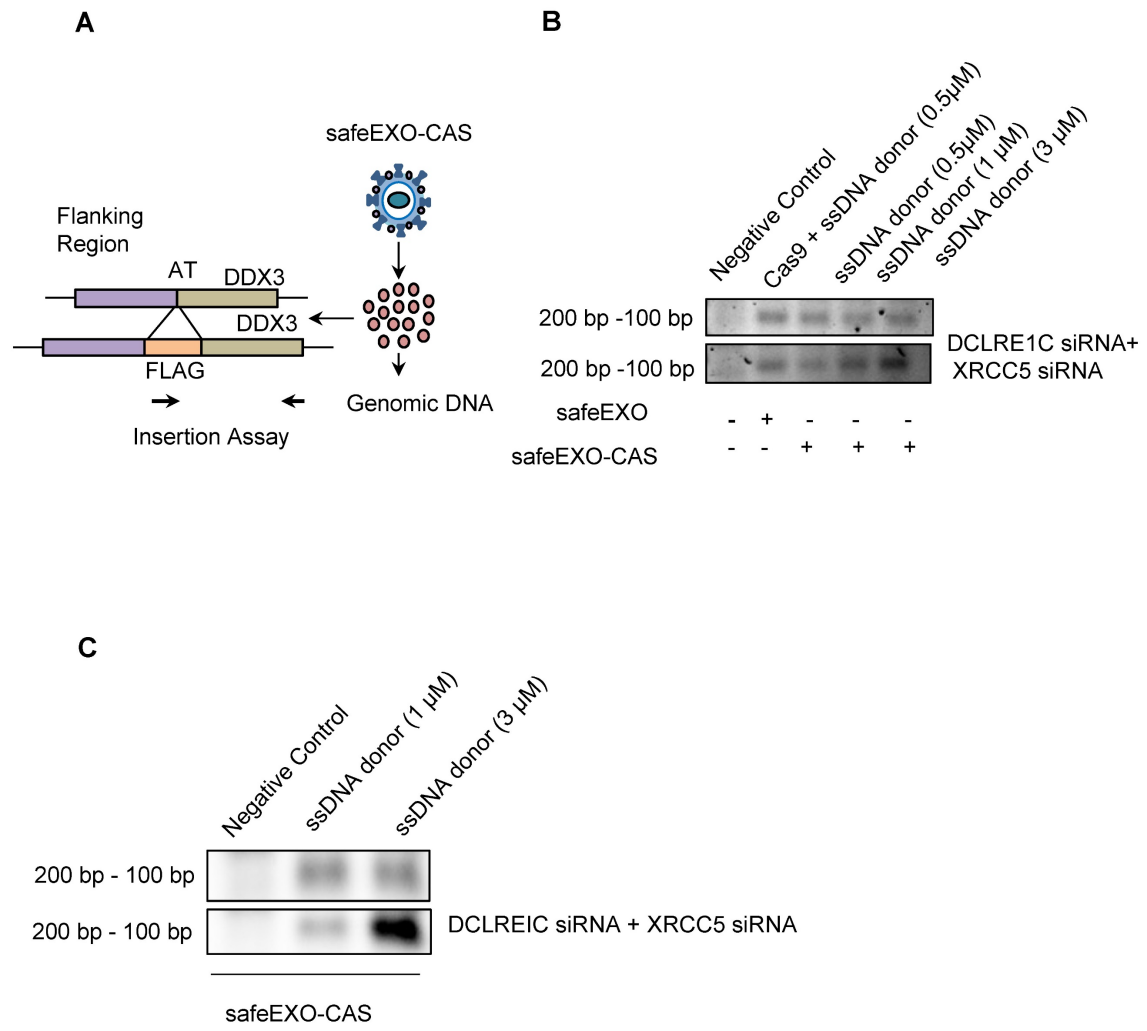
SafeEXO-CAS-mediated homology-directed repair (HDR) editing. **A** Schematic representation of sEVs HDR experimental plan in HEK293T cells. **B** SafeEXO-CAS sEVs co-loaded with sgRNA targeting human DDX3 at its start codon and donor ssDNA encoding the FLAG-tag protein surrounded by homology arms to the DDX3 start codon (top). SafeEXO-CAS sEVs were also co-loaded with siRNAs targeting DCLRE1C and XRCC5 to prevent NHEJ (bottom). HEK293T cells were treated with sEVs containing three different concentrations (0.5uM-3uM) of ssDNA donor template. The FLAG-tag insertion was confirmed using the forward primer from FLAG-tag and reverse primer from the DDX3 locus. **C** HEK293T cells were treated with safeEXO-CAS sEVs co-loaded with sgRNA targeting human GFP at its start codon and a ssDNA template encoding the FLAG-tag protein and bearing homology arms to the GFP start codon (top). SafeEXO-CAS sEVs were also co-loaded with siRNAs targeting DCLRE1C and XRCC5 to prevent NHEJ (bottom). HEK293T cells were treated with sEVs containing two different concentrations (0.1uM-3uM) of ssDNA donor template. The FLAG-tag insertion was confirmed using the forward primer from FLAG-tag and reverse primer from GFP locus.

Precise insertion of genetic material (also known as knock-in) using CRISPR-Cas9 could be accomplished through HDR. HDR is significantly more challenging as a therapeutic modality compared to NHEJ due to decreased efficiency and the added complexity of the payload, which in addition to the Cas9/sgRNA, also must include a donor DNA template. HDR induction was evaluated in target cells after exposure to safeEXO vehicles loaded with the complex payloads needed for HDR, including sgRNA that targeted a locus close to the ATG start codon of the human DDX3 gene along with a template single-stranded DNA oligomer encoding the FLAG-tag reporter flanked with 46 nucleotide (nt) homology arms targeting the same region as the sgRNA **(Figure 5A)**. Cells were exposed to safeEXO-CAS loaded with the sgRNA and ssDNA donor. Cells were passed 5 times after sEV exposure before assessing HDR insertion efficiency by PCR. Cells treated with safeEXO-CAS loaded with sgRNA and ssDNA demonstrated incorporation of the FLAG-tag at the DDX3 locus, proving successful HDR gene editing. A dose-dependent increase in the editing efficiency was observed when ssDNA donor concentration was increased from 0.5µM to 3µM **(Figure 5B)**. To increase HDR efficiency, we co-loaded safeEXO-CAS/sgRNA/ssDNA vehicles with additional siRNAs that target DCLRE1C and XRCC5 genes to prevent non-homologous end joining, resulted in an increase in the insertion of ssDNA to the genome and higher efficiency of HDR **(Figure 5B)**. HDR genomic editing was independently confirmed in a second cell line and target by exposing HEK-GFP cells with safeEXO-CAS containing sgRNA targeting GFP together with a ssDNA template. HDR was confirmed using a genetic insertion assay **(Figure 5C)**, showing safeEXO-CAS could co-deliver ssDNA, sgRNA, and siRNAs (DCLRE1C and XCRCC5) to efficiently mediate gene insertion in a dose-dependent manner **(Figure 5C)**. These data demonstrate the ability of our platform to simultaneously co-deliver highly complex payloads, including CAS, ssDNA, sgRNAs, and siRNAs (DCLRE1C and XCRCC5) to enable efficient HDR-mediated gene insertion, which cannot be efficiently accomplished with other means of delivery.

### SafeEXO-CAS mediated genomic editing minimizes the off-target effect

Initial safety of safeEXO-CAS genome editing was tested looking for cellular inflammatory responses and off-target editing. No significant induction of any cytokines was detected after treatment with safeEXO-CAS **(Figure S6).** Off-target editing of safeEXO-CAS was compared to plasmid delivery methods using the standard mismatch tolerance assay utilizing gRNAs with different base mismatches compared to the targeted sequence. Our results demonstrated that safeEXO-CAS genome editing resulted in lower off-target editing compared to plasmid-based gene editing **(Figure S7)**.

### Targeting safeEXO vehicles to specific biomarkers *in vitro* and *in vivo*

**Figure 6:**
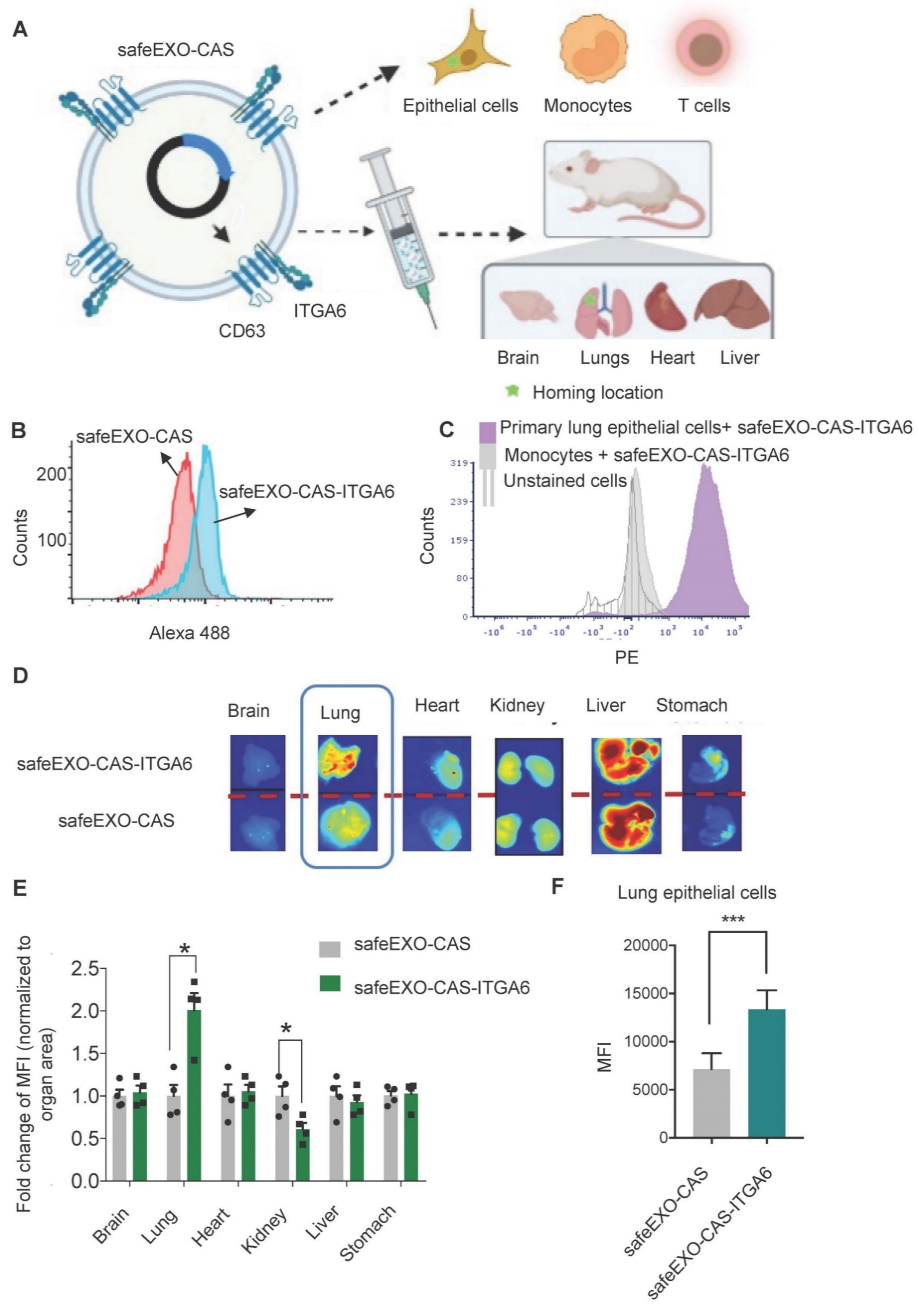
**A** Schematic representation of *in vitro* and *in vivo* safeEXO-CAS-ITGA6 lung targeting. **B** Flow cytometry analysis using an ITGA6 (CD49f) antibody of safeEXO-CAS-ITGA6 and untargeted safeEXO-CAS. **C** Flow cytometry analysis of the fluorescently labeled safeEXO-CAS-ITGA6 uptake by primary lung epithelial cells, monocytes and unstained cells after 6h coculture. **D** ExoGlow labeled safeEXO-CAS-ITGA6 sEVs or untargeted safeEXO-CAS sEVs uptake by different mouse organs (brain, lung, heart, kidney, liver and stomach) using optical imaging of organs 15 min after sEVs injection. **E** Fold change of fluorescence intensity normalized to organ area from different organs (n=8 total). **F** Percentage of sEVs uptake was quantified by flow cytometry in lung epithelial cells (EpCAM^+^) of mice receiving safeEXO-CAS-ITGA6 or untargeted safeEXO-CAS control sEVs (n=6 per group). Data are represented as mean ± standard deviation (SD). MFI, mean fluorescent intensity, *p ≤ 0.05, Student's T test was used for pairwise comparison.

To further optimize the safeEXO-CAS sEVs for *in vivo* therapeutic genomic editing, we tested the potential to engineer them with targeting moieties. SafeEXO-CAS producer cells were engineered to express the integrin alpha chain alpha 6 (ITGA6) on the surface of their sEVs to increase potential lung targeting, as biodistribution studies of circulating sEVs indicated that expression of ITGA6 provides homing to the lung 39. To guide the targeting moiety to the sEV surface, producer cells were transfected with cDNA encoding a CD63-ITGA6 protein **(Figure 6A)**. SafeEXO-CAS-ITGA6 sEVs were collected from these producer cells, and flow cytometry quantification of ITGA6 (CD49f) showed a significant increase of ITGA6 on the sEV surface **(Figure 6B)**. ITGA overexpression on SafeEXO-CAS-ITGA6 sEV did not induce any significant invasion and proliferation in 2 different lung cancer cell lines **(Figure S8)**. In a competitive co-culture between targeted lung epithelial cells and non-targeted primary monocytes, safeEXO-CAS-ITGA6 sEVs demonstrated higher uptake in the lung epithelial cells **(Figure 6C)**.

To test *in vivo* targeting, biodistribution studies were performed on healthy mice after injection of NIR-labeled safeEXO-CAS-ITGA6 sEVs versus control NIR-labeled untargeted safeEXO-CAS sEVs. SafeEXO-CAS-ITGA6 sEVs demonstrated higher uptake in the lung **(Figure 6D-E)** and corresponding lower uptake in the kidney compared to control untargeted safeEXO-CAS sEVs. Flow cytometry of lung epithelial cells (EpCAM^+^) showed an increased presence of sEVs in the lung epithelial cells isolated from mice that received safeEXO-CAS- ITGA6 sEVs compared to the controls **(Figure 6F)**. These data indicate that expression of ITGA6 on the surface of safeEXO-CAS sEVs increases their uptake in lung epithelial cells. The liver demonstrated intense uptake of both targeted and untargeted sEVs, which has been shown by many groups, as the liver plays a primary role in clearing EVs from circulation. However, only the specific binding to the lung by targeted safeEXO-CAS-ITGA6 sEVs demonstrated successful genomic editing, in contrast to the liver.

### SafeEXO-CAS-ITGA6 can mediate successful genome editing in lung

**Figure 7:**
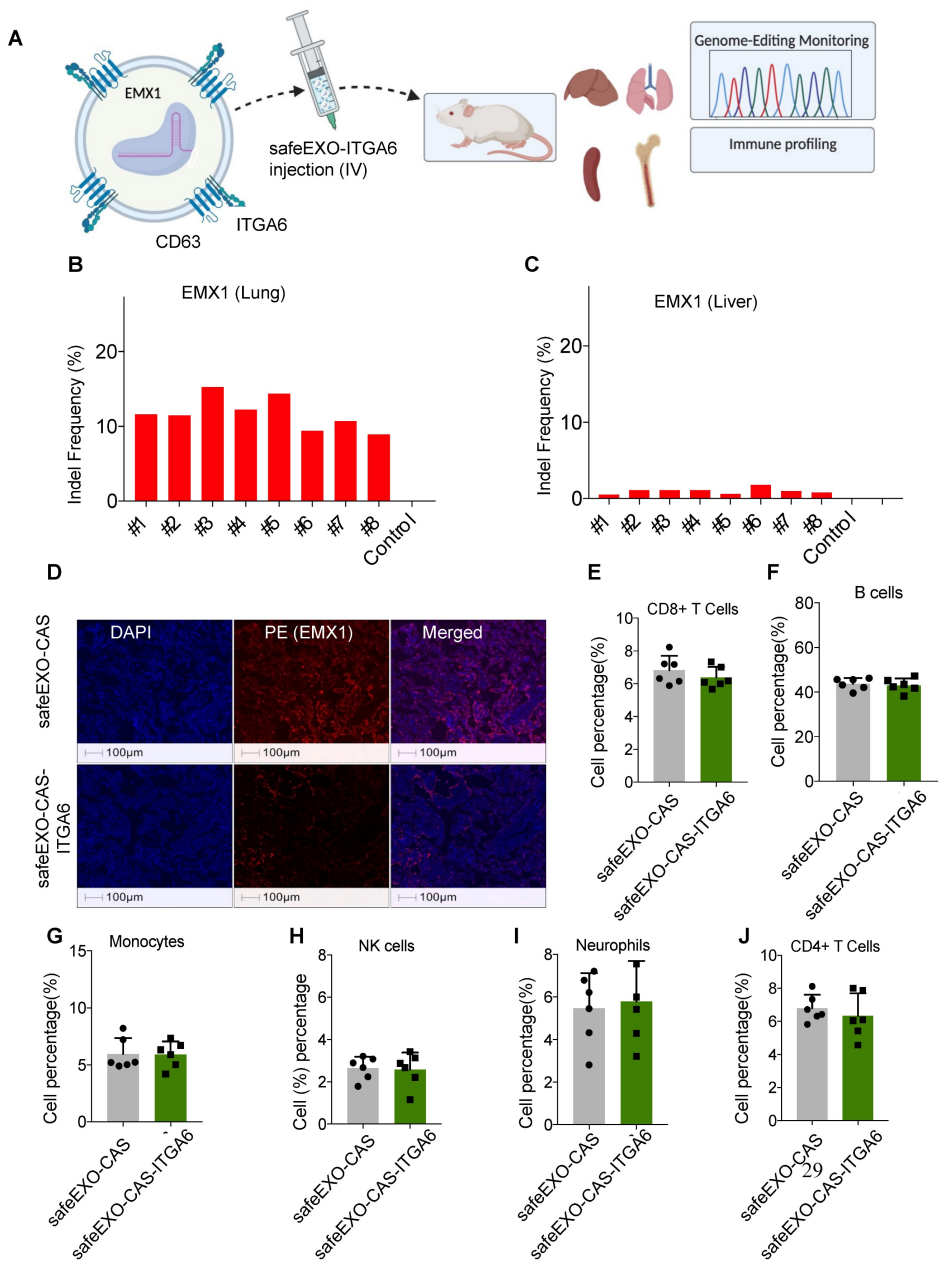
**A** Schematic representation of safeEXO-CAS-ITGA6 mediated EMX1 editing *in vivo*. **B** Percentage of indel frequency in EMX1 in lung and **C** liver (n=8 per group). **D** Representative immune fluorescence of EMX1 expression in lung tissue of safeEXO-CAS-ITGA6 and safeEXO-CAS. **E-J** Percentage of immune subtypes frequency in CD8^+^T cells, B cells, monocytes, NK cells, neutrophils, and CD^+^4 T cells (n=6 per group). Data are represented as mean ± standard deviation (SD).

Genomic editing was tested by loading safeEXO-CAS- ITGA6 sEVs with 3 sgRNAs designed to disrupt the reading frame of EXM1 through indels caused by NHEJ. sgRNA-loaded safeEXO-CAS-ITGA6 sEVs or control vehicle (unloaded safeEXO-CAS) were systemically injected into mice, which were analyzed for genome editing two weeks after injection** (Figure 7A)**. All mice injected with safeEXO-CAS-ITGA6 loaded with sgRNA targeting EMX1 displayed significant editing in the lung (between 7% and 16% efficiency) **(Figure 7B)** compared to the no editing detected in lungs of control mice. In contrast, no significant editing was seen in other organs, including in the liver **(Figure 7C)**, despite the high localization of the sEVs in the biodistribution studies (**Figure 6D**). These data indicate that the untargeted liver uptake is likely due to the sEVclearance function of the liver and does not indicate functional targeting. Consistently, when lung tissue from mice injected with safeEXO-CAS-ITGA6, which was loaded with sgRNA targeting EMX1, was examined through immunofluorescence, there was a notable reduction in EMX1 expression compared to the lung of control mice **(Figure 7D).**

### Initial toxicology studies after safeEXO mediated genomic editing

To assess the adverse effects of our safeEXO-CAS-ITGA6 genomic edition vehicle, we observed clinical morbidity and performed immune profiling and plasma toxicology studies on the EXM1-edited mice. There were no associated signs of morbidity and immune profiling of safeEXO-CAS-ITGA6 treated mice and control mice did not show any detectable changes in the frequencies of immune cells (CD8 T cells, CD4 T cells, NK cells, monocytes, B cells, and neutrophils) in the spleen **(Figure 7E-J)** and bone marrow** (Figure S9)**. Blood biochemical and toxicological panels did not reveal any systemic adverse effects of safeEXO-CAS-ITGA6 sEVs including changes in the kidney and liver function indicators including ALT, AST, creatinine, and blood urea nitrogen (BUN test) **(Table S1 and Figure S10)**.

## Discussion

The results presented in this study demonstrate the potential of the safeEXO platform as a novel, non-invasive method to deliver RNAi and CRISPR-based gene editing components for high-efficiency delivery and genomic editing. We showed that safeEXO-CAS sEVs can effectively deliver highly complex payloads, including sgRNA, ssDNA, and siRNAs, a novel strategy that we demonstrate can block NHEJ and facilitate HDR-based gene insertions, which is not feasible with other delivery modalities. Furthermore, we engineered the safeEXO-CAS sEVs to express ITGA6 on their surface as an example of tissue/biomarker targeting and showed their potential to facilitate efficient *in vivo* gene editing in mouse lungs at 10-15% efficiency after a single injection.

Our findings suggest that safeEXO-CAS sEVs have a wide range of potential applications, from *in vitro* to preclinical studies, and potentially future clinical exploration. SafeEXO-CAS sEVs could be used to study gene functions and develop novel therapies for different diseases ranging from monogenetic disorders to cancer. As safeEXO-CAS sEVs were non-immunogenic, they have the potential to be used *in vivo* to target specific organs or tissues with CRISPR components without eliciting an immune response. For example, our prototype targeted safeEXO-CAS sEVs displaying integrin alpha-6 (ITGA6) on their surface could be useful for the treatment of lung diseases, as our results demonstrate they can deliver functional CRISPR components to the lungs and mediate targeted efficient genome editing preferentially to targeted tissue and not the liver.

The therapeutic potential of sEVs has been explored to discover novel treatments with improved specificity and efficiency for different organs such as the brain. sEVs derived from cell types native to the central native system, such as microglia, macrophages, and brain endothelial cells, are capable of bypassing the blood-brain barrier, facilitating brain-targeted drug delivery, but these native cell-derived exosomes exhibited a high off-target rate 40, 41. Further genetic modification of sEVs has been made to tackle more effective targeted delivery. A range of proteins expressed by sEVs were genetically modified or fused to viral proteins to enhance brain-specific delivery of RNAs for genetic therapies 41. It was reported that lysosome-associated membrane glycoprotein 2b (Lamp2b), a sEV surface protein, was fused to a 29-amino-acid peptide derived from rabies virus glycoprotein that recognized nicotinic acetylcholine receptor. This engineered protein facilitated the delivery of siRNA targeting BACE1 to the brain and successfully knocked down BACE1 42. Recently, it has been shown that the electroconductive hydrogel loaded with bone marrow stem cells-derived exosomes facilitates the attachment and migration of Shwann cells *in vitro*. This engineered hydrogel promoted neural regeneration and functional restoration, while the bone marrow stem cells-derived exosomes participated in NFκB pathway and regulated M2 macrophage polarization, thus ameliorating inflammatory pain 40.

The mesenchymal stem cells-derived sEVs showed promising potential for novel treatments of osteoarthritis, given their high biocompatibility, low immunogenicity, and better ability to cross biological barriers compared to MSCs 43. Infrapatellar fat pad (IPFP)-derived MSCs promoted the proliferation of chondrocytes and synthesis of extracellular matrix and reduced expression of catabolic factors to prevent cartilage damage and relieve walking disability 44. Xu et al. reported anti-mRNA delivery by red blood cell-derived engineered EVs to osteoclasts as a potential novel treatment of osteoporosis 45. The red blood cell-derived engineered EVs were genetically modified to express a bi-functional fusion protein of TRAP-binding protein and a viral protein CP05 to guide the targeted delivery to osteoclast 45.

The successful generation of sEV-based drug delivery platforms for the targeted delivery of CRISPR/Cas offers numerous advantages over existing viral or non-viral vectors. These engineered sEVs are biocompatible, non-immunogenic, and non-toxic, even in repeated *in vivo* injections. Furthermore, their lipid bilayer protects the protein and RNA cargos from enzymes such as proteases and RNases, allowing for long circulation times. Finally, sEVs carrying bacterial-derived Cas machinery have been demonstrated to exhibit an increased capacity to escape degradation or clearance by the immune system compared with existing CRISPR delivery vectors. The successful delivery of functional CRISPR/Cas systems using sEVs, as demonstrated here, provides a promising and powerful platform for gene editing *in vitro* and *in vivo*. Our findings confirm the principle that safeEXO-CAS sEVs are competent in performing precise gene editing tasks and demonstrate their lung-targeting capabilities shortly after administration. To fully understand the broader implications of safeEXO-CAS usage, further investigations into its biodistribution over prolonged periods are necessary.

This work provides a promising step towards achieving effective, targeted delivery of CRISPR/Cas systems for gene editing in a scalable and safe manner. Further research is needed to identify the optimal combination of nucleic acid and Cas9 components, and sEV dosing in the context of disease. Taken together, this work provides an exciting platform for future development of sEV-based gene editing applications. If successful, the safeEXO-CAS platform could impact precision medicine by providing an effective and scalable gene editing therapy with minimal off-target effects. This would enable personalized therapeutics for a wide range of diseases caused by genetic mutations, leading to more targeted treatments that improve patient outcomes.

## Methods

### Cell culture, transfections, and cloning

THP-1, Cas9 expressing THP-1 monocytes, A549, NCI-H2030, and KRAS 4B wild type cells were cultured in RPMI-1640 and HEK293T in DMEM, supplemented with 10% Fetal Bovine Serum (FBS) (Corning #35-011-CV) containing 1X Penicillin and Streptomycin mix (100X penicillin (10,000IU) and streptomycin (10,000µg/ml) mix) (Corning #30-002-CI). All cell lines were cultured and incubated at 37 °C with 5% CO_2_. Cas9-expressing THP-1 monocytes were transfected with fluorescently labeled sgRNA against Drosha (IDT), ALIX (IDT), and hnRNPA2B1 (IDT). Briefly, 60 pmol of Alt-R CRISPR-Cas9 gRNA (IDT) per 1E6 cells per target were transfected using lipofectamine RNAiMAX following the manufacturer's established protocol. After 24h, individual cells were sorted and individual knockout clones' selection was confirmed using western blot. All experiments involving sEVs' isolation were performed in media supplemented with exosome-depleted fetal bovine serum (Gibco #A27208-03). For the generation of Drosha, Alix, and hnRNPA2B1 knockout cells, cells were transfected with 2sgRNAs against each gene and screened by western blot as described below to identify the knockout clones. Mice ITGA6 was cloned into the MCS of CD63-pEGFP C2 vector backbone from Addgene (Plasmid# 62964) with restriction site SacI and EcoRI. ITGA6_mice_forward: 5′ AGAGAGCTCCATGGCGGTCGCGGGCCAGTTGT 3′; ITGA6_mice_reverse: 5′ AGAGAATTCTCCTCCTCCTCCTCCTGCATCGGAAGTAAGCCTCTCTTTATCAGA 3′.

### Collection and purification of sEVs

In a T75 or T25 flasks producer cells of sEVs were seeded and 24 h later transfected with 24 µg total of plasmids or vectors if indicated. After 12 h of transfection, the cell culture medium was replaced. After 72 h of transfection, the media was collected and subjected to sequential spins: a low-level spin at 800 × *g* for 5 mins to remove cells, followed by 2000 × *g* for 20 mins to remove of cell debris. There are reports analyzing different methods of EVs isolation and for our study we chose the Exoquick-TC based method, yielding pure and intact EVs suitable for functional assays and their applications 46. Briefly, ExoQuick-TC ULTRA (for cell culture medium) (System Biosciences) was used according to the manufacturer's protocol. The ExoQuick was added followed by an incubation overnight at 4°C following the protocol recommended for the manufacturer. After incubation, the precipitate was spun down (3000 × *g*, 10 min at 4°C), sEVs were resuspended in buffer and added to the purification columns and sEVs were eluted in volume of 500 μl elution buffer per isolation. The final volume was passed through a 0.22-micron filter (Millipore Sigma). The sEV isolated fraction and EV-depleted fraction were frozen and stored at -80°C until further use.

### sEVs surface marker analysis

The sEVs were incubated with capture beads for CD81 (ab239687, ABCAM) and CD63 (ab239686, ABCAM) overnight at 4 °C, as recommended by the manufacturer. Primary detection antibody mixture (CD49f, CD63 and CD81 conjugated to fluorescent dyes) was added to the mixture of samples and beads (1:25). Samples were mixed gently and incubated for 1h at 4°C. Samples were washed 2 times by assay buffer and resuspended in 350ul FACS buffer. Samples were run on a BD FACSAria II and data was analyzed using FCS Express Analysis Software (De Novo Software, Pasadena, CA).

### Protein quantification and immunoblot analysis

sEVs along with whole cell pellets were lysed in RIPA Lysis and Extraction Buffer (Thermo Fisher #89900). Protein concentration was determined by a Pierce BCA Protein Assay Kit (Thermo Fisher #23227) as per manufacturer's instructions.

sEVs and cell lysates were lysed in RIPA buffer and run on a 15% polyacrylamide gel with equal amounts of protein loaded (100 μg). Proteins were transferred to nitrocellulose membrane (Bio-Rad #1620115) and then blocked for 1 hours in 1X TBS 1% Casein Blocker (blocking buffer) (Bio-Rad #1610782).

The following primary antibodies were used: Exosome-anti-CD81 antibody (Invitrogen #10630D), Exosome-anti-CD63 antibody (Invitrogen #10628D), Drosha Antibody (Invitrogen #PA5-79927), hnRPA2B1 Polyclonal Antibody (Invitrogen #PA5-34939), Cas9 Antibody (10C11-A12) (Invitrogen #MA1-202), Alix Antibody (3A9) (Invitrogen #MA1-83977). All primary antibodies were used at a dilution of 1:1000 in the blocking buffer and were incubated overnight. For detection, secondary goat anti-mouse IgG (H+L)-HRP Conjugate antibody (Bio-Rad #170-6516) and goat anti-rabbit IgG (H+L)-HRP Conjugate antibody (Bio-Rad #170-6515) was used for 2 hours at a dilution rate of 1:3000 in blocking buffer. The immunoreactive bands were visualized by a Clarity Max™ Western ECL substrate (Bio-Rad #1705062) according to the manufacturer's protocol and an iBright Imaging system (Thermo Fisher Scientific #CL1000).

### Ribonucleoproteins (RNP) complex formation and sEVs loading

The gRNAs and Cas9 enzyme were combined in equimolar amounts in 1XDPBS and incubated at room temperature for 5-10 min for RNP complex formation. Isolated sEVs were loaded using Exo-Fect^TM^ Exosome Transfection Kit (Cat EXFT10-1), according to the manufacturer's instructions. Briefly, sEVs were resuspended in 1XDPBS followed by mixing with Exo-Fect solution and loading cargo material (siRNA, gRNA, gRNA-Cas9 as RNP). The mix is further incubated at 37ºC in a shaker for 10 minutes and then immediately placed on ice. Afterward, the transfected sEVs were isolated by adding ExoQuick-TC reagent, based on the recommended protocol by the manufacturer. After removing the supernatant, the pellet comprising transfected sEVs was resuspended in DPBS to be used in experiments.

### Invasion assay

To conduct invasion assays, a cell suspension measuring 220 μL was introduced into the upper compartment of a Transwell chamber, specifically designed for 24 wells. The upper compartment's surface was pre-coated with Matrigel at a concentration of 1 mg/mL, sourced from Corning, the lower compartment was filled with 500 μL of RPMI-1640 medium enriched with 10% fetal bovine serum (FBS). Following the incubation period, cells adhered to the filter membrane were fixed using a 4% formaldehyde solution for a duration of 15 minutes. Subsequently, these cells were stained with a 0.5% solution of crystal violet to facilitate their visualization and enumeration under a light microscope.

### MTT assay

A549 or NCI-H2030 cells were plated in 96-well plates. After 24 h, 25 ug of safeEXO-CAS and safeEXO-CAS-ITGA6 were added to the culture media. 3-(4,5-Dimethylthiazol-2-yl)-2,5-diphenyltetrazolium bromide assay was performed using the Vybrant® MTT Cell Proliferation Assay Kit, as described by the manufacturer. The absorbance of the samples was measured at 540 nm using a microtiter plate reader. Experiments were assayed in triplicate.

### Mice and *in vivo* biodistribution studies

C57BL/6J mice (JAX stock #000664, Jackson Laboratory) (6-8 weeks old) were maintained following the Guide for the Care and Use of Laboratory Animals. Animal housing follows a 12 h light/12 h dark cycle, wherein the temperature is maintained between 68-79 ˚F. Humidity was maintained between 30-70 percent. All experiments were performed according to the guidelines of the Institutional Animal Committee of the Columbia University.

sEVs were labeled with ExoGlow (EXOGM600A-1, SBI) as described by the manufacturer. Briefly, 1µl of ExoGlow was added to 100µg of sEVs in 200µl 1X PBS and incubated at room temperature for 1hr. Labeled sEVs were mixed with 63µl of ExoQuick-TC and incubated overnight at 4°C followed by centrifugation at 13,000 *g* for 10 mins. The resulting pellet containing labeled sEVs was resuspended in 1X PBS buffer. Further, 100 µg of sEVs were administered into mice in a total of 100 µl volume via IV injection. 15-30 minutes after sEV injection, mice were euthanized using an overdose of isoflurane anesthesia. The animals and organs (brain, lung, heart, liver, and spleen) were imaged using an IVIS spectrum bioimaging device (PerkinElmer). The intensity of the fluorescence was calculated using the ImageJ and normalized to the controls. Blood was collected for toxicological and biological assessments.

### Immunofluorescent staining

Immunofluorescence staining targeting EMX1 was performed following a standard protocol for formalin-fixed paraffin-embedded (FFPE) samples. The process began with removing the paraffin from the slides, which were then submerged in a plastic container containing citrate-based antigen retrieval (AR) solution. This solution was heated to a boil for 1 minute at 100°C, followed by microwaving the sections for 15 minutes at 75°C. After cooling in the AR solution for 15 minutes at ambient temperature, the slides were washed with deionized water and Tris-buffered saline with 0.1% Tween 20. A Tris-HCl buffer with 0.1% Tween was applied for 10 minutes at room temperature to stabilize proteins and minimize nonspecific background staining. The slides were subsequently incubated with an EMX1-specific antibody (Invitrogen, Catalog # PA5-35373) at a 1:300 dilution for 2 hours. Following this, the slides underwent washing steps before being incubated with rabbit anti-mouse secondary antibodies (Rabbit IgG (H+L) Cross-Adsorbed PE Secondary Antibody, Catalog # P-2771MP) for 10 minutes at room temperature, with additional washes in TBST. After three final washes in deionized water, DAPI was used for nuclear counterstaining for 5 minutes, and the slides were then covered with VECTASHIELD Antifade Mounting Medium (Vectorlabs) to preserve fluorescence. Visualization was achieved using an EVOS microscope.

### Nanoparticle tracking analysis (NTA)

The concentration and size of sEVs in cultured media were identified by a NanoSight NS300 system (NanoSight, Amesbury, UK) supplied with a fast video capture and Nanoparticle Tracking Analysis (NTA) software. Before performing the experiments, the instrument was calibrated with 100 nm polystyrene beads (Thermo Scientific, Fremont, CA, USA). The samples were imaged three times each for 30s at 25°C. NTA software processed the video captures and measured the associated particle concentrations (particles/ml), size distributions (in nanometer), and intensities (arb. units) of the samples.

### Transmission electron microscopy

Transmission Electron Microscopy by Negative Staining 5 μl of purified sEVs was added onto glow discharged (PELCO easiGlow, Ted Pella. Inc., Redding, CA) carbon-coated 400 mesh copper grid (Electron Microscopy Sciences, Hatfield, PA), and stained with 1% aqueous uranyl acetate (Polysciences, Inc, Warrington, PA). Stained grids were imaged under JEOL1400 Flash electron microscope (Japan) and photographed with a Gatan Rio 16 camera (Gatan Inc. Pleasanton, Pleasanton, CA).

### sgRNA design and sequences (+PAM)

sgRNAs targeting *DDX3*, *GFP*, *EMX1* were designed using IDT software.

Human *DDX3*: 5′ AGGGATGAGTCATGTGGCAGtgg 3′

Human *EMX1*: 5′ GAGTCCGAGCAGAAGAAGAAggg 3′

Mouse *EMX1_SG1*: 5′ CAAGCGACGTTCCCCAGGACggg 3′

Mouse *EMX1_SG2*: 5′ CCAAGGATGGTGGCACCGGCggg 3′

Mouse *EMX1_SG3*: 5′ GGCAGGGAAGCCACTCACGAagg 3′

*GFP*: 5′ CGGCCATGATATAGACGTTGtgg 3′

### T7 endonuclease assay

Genomic DNA was extracted from cells using the Quick-DNA Miniprep Kit (Zymo Research # D3024). 40ng of genomic DNA was then used for PCR amplification for 25µl reaction. Further, in order to form heteroduplexes Alt-R Genome Editing Detection Kit (IDT # 1075932) was used. Briefly, 10µl PCR products were added with T7EI reaction buffer (10X) and 6µl H20 to a final volume of 18µl. The reaction was heated and cooled according to the following procedure: heated at 95°C for 10 min, ramping from 95-85°C at a ramp rate of -2°C/sec (ramp1), then ramping from 85-25°C at a ramp rate of -0.3°C/sec (ramp2). For T7EI digestion, 2µl of T7 endonuclease I(1U/µl) was added to the heteroduplexes. The reaction was incubated at 37°C for 60 minutes. Samples were finally run on a 2.5% agarose gel. For T7 endonuclease assay the primers used were:

GFP_F: GCAAGGGCGAGGAGCTGTTCAC

GFP_R: AGGTAGTGGTTGTCGGGCAGCAG

EMX1_T7endoF: TTCTCTCTGGCCCACTGTGTCCTC

EMX1_T7endoR: AGCCCATTGCTTGTCCCTCTGTCAATG

### sEV-mediated homology-directed repair using ssDNA

sEVs were loaded with different concentrations of ssDNA donor template containing FLAG-tag (5′-ACTCGCTTAGCAGCGGAAGACTCCGagTTCTCGGTACTCTTCAGGGATGGA CTACAAGGACGACGATGACAAGagTCATGTGGCAGTGGAAAATGCGCTCGGGCTGGACCAGCAGGTGA-3') targeting AUG codon of DDX3 at 0.5 µM, 1 µM and 3 µM with or without addition of DCLRE1C siRNA (cat# AM16708) and XRCC5 siRNA (cat# AM16708). 8X10^4^ THP-1 (0.5 µM, 1 µM and 3 µM of ssDNA) or HEK293T (1 µM and 3 µM of ssDNA) cells were treated with sEVs. cells were then collected for the genomic DNA isolation and PCR. Cells were checked for the insertion using the following primers: FLAG-tag-Forward 5′-GACTACAAGGACGACGATGACAAG-3′ and DDX3-Reverse2 5′-CGCCATTAGCCAGGTTAGGT-3′.

### High-throughput sequencing of Emx1

Genomic DNA was extracted from sEVs-treated cells using the Genomic DNA extraction kit (Zymo Research, Irvine, CA). 40 ng of genomic DNA was then used for PCR amplification using primers specific for EMX1 (EMX1-Forward 5′- GGCCCAGGTGAAGGTGTGGTT -3′ and EMX1-Reverse 5′- GGTTGCCCACCCTAGTCATTGGA -3′). Obtained PCR products were gel purified and sequenced at the MGH Center for Computational and Integrative Biology (CCIB) at Boston, Massachusetts. The CRISPR editing efficiency was identified using CRISPResso2 47.

### IV injection of safeEXO-CAS

Genomic DNA from each mouse (treated either by control or lung targeting safeEXO-CAS) was extracted from lung and liver. Following this, a PCR was performed on 40 ng of gDNA template, the PCR was performed using the following primers: EMX1_sgRNA2_Forward: TTAGGGCTCTCGCACGCCCCTC, EMX1_sgRNA2_Reverse: TGGTTCATGGCCTCTGGGAACACCA, EMX1_sgRNA3_Forward: TGCACACCCCGCACGGCGGCA, EMX1_sgRNA3_Reverse: CCTGGAAGCGGTGGCCAAAGAAGCGA, to amplify the EMX1 amplicon (95 °C -5 min, 35 cycles (95 °C -30 sec, 60 °C -30sec, 72 °C -45sec), 72 °C -5 min). Amplicons were run on 1% Agarose gel and purified after excision. Further, PCR amplified products were analyzed by sanger sequencing (GENEWIZ, NJ) electropherograms and TIDE analysis.

### Generation of single-cell suspensions and flow cytometry analysis

Single-cell suspensions of the spleen and bone marrow were prepared for analysis. Spleens were homogenized through a 70 μm cell strainer, while bone marrow was flushed into a falcon tube with a syringe. Erythrocytes in the suspension were lysed by ACK lysing buffer for 4 minutes, and the reaction was then stopped with PBS 0.1% BSA. Finally, the suspension was passed through a 30 μm cell strainer. Lung tissue was dissociated using a Miltenyi dissociation kit. The lung epithelial cells were harvested by positive selection using EpCAM (cat#130-105-958, Miltenyi), the pan-epithelial marker based on the manufacturer's protocol. Flow cytometry for identification of cell counts and mean fluorescent intensity of immune cells was performed on a BD FACSAria II. Immune cell populations were defined on live lymphocytes with CD45 and Zombie Aqua™ (cat# 423101) and CD4^+^ T cells (TCRβ^+^CD4^+^), CD8^+^ T cells (CD4^-^CD8^+^), NK (TCRβ^-^NK1.1^+^), neutrophils (Ly6G^+^CD11b^+^), monocytes (TCRβ^-^Ly6G^-^CD11b^+^Ly6C^-^), B cells (TCRβ^-^CD19^+^). Flow cytometry data was analyzed using FCS Express Analysis Software (De Novo Software, Pasadena, CA). The following antibody clones were used for this study: CD45 (Alexa Fluor 488, 30-F11, BioLegend), TCRβ (BUV737, H57-597, BD), CD8a (Alexa 700, 53-6.7, BioLegend), CD4 (BV605, RM4-5, BioLegend), NK-1.1 (Brilliant Violet 650, PK136, BioLegend), Ly6G (BUV395, 1A8, BD), Ly6C (BV786, HK1.4, BioLegend), CD11b (BV421, M1/70, BioLegend), CD19^+^(PE, 4G7, BioLegend).

### Statistical analysis

Data presented as mean ± standard deviation (SD) or mean ± standard error of the mean (SEM) as indicated. Data analysis was performed by GraphPad Prism 6.01 (GraphPad, USA) and R software (V 3.6). Differences between the two groups were tested using the student's *t*-test. Differences among multiple groups were analyzed using one-way ANOVA followed by Dunnett's post hoc comparisons. *P* value <0.05 was considered significant. Experiments were repeated at least two times with at least three replicates. Statistical significance was annotated as follows: *P < 0.05, **P < 0.01, ***P < 0.001, ***P<0.0001.

## Supplementary Material

Supplementary figures and table.
