# Supplementary material for: Small extracellular vesicles (sEVs)-based gene delivery platform for cell-specific CRISPR/Cas9 genome editing
Source: Theranostics. 2024 Apr 28;14(7):2777–93. doi: 10.7150/thno.92133 (PMC11103490; doi:10.7150/thno.92133)
Supplement: Supplementary file 1 — Supplementary figures and table. [file thnov14p2777s1.pdf]

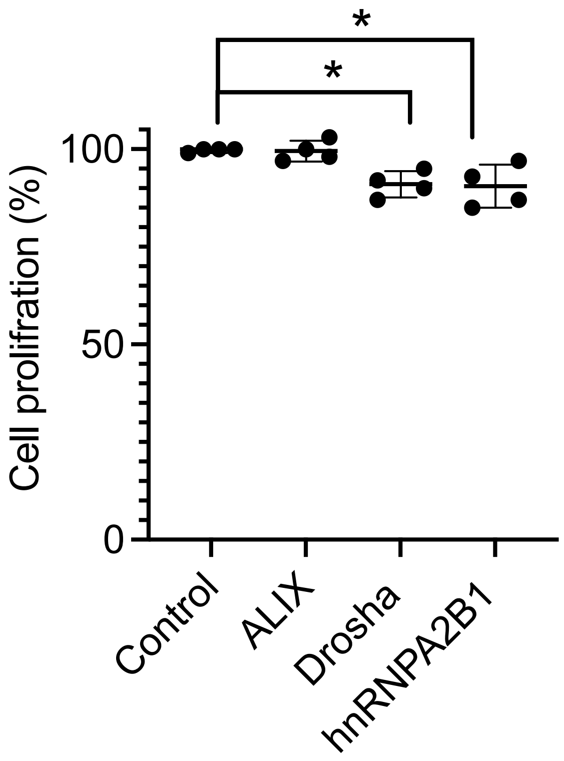

Figure S1- Dot plot showing the change in cell density following 48 hours of control siRNA, ALIX or Drosha or hnRNPA2B1 siRNAs. Scatter plots represent a mean ± standard deviation (SD). n = 4 per group. <0.0001, One-way ANOVA was use for comparison of multiple group and Student’s T test was used for pairwise comparison. *$p < 0.05$.

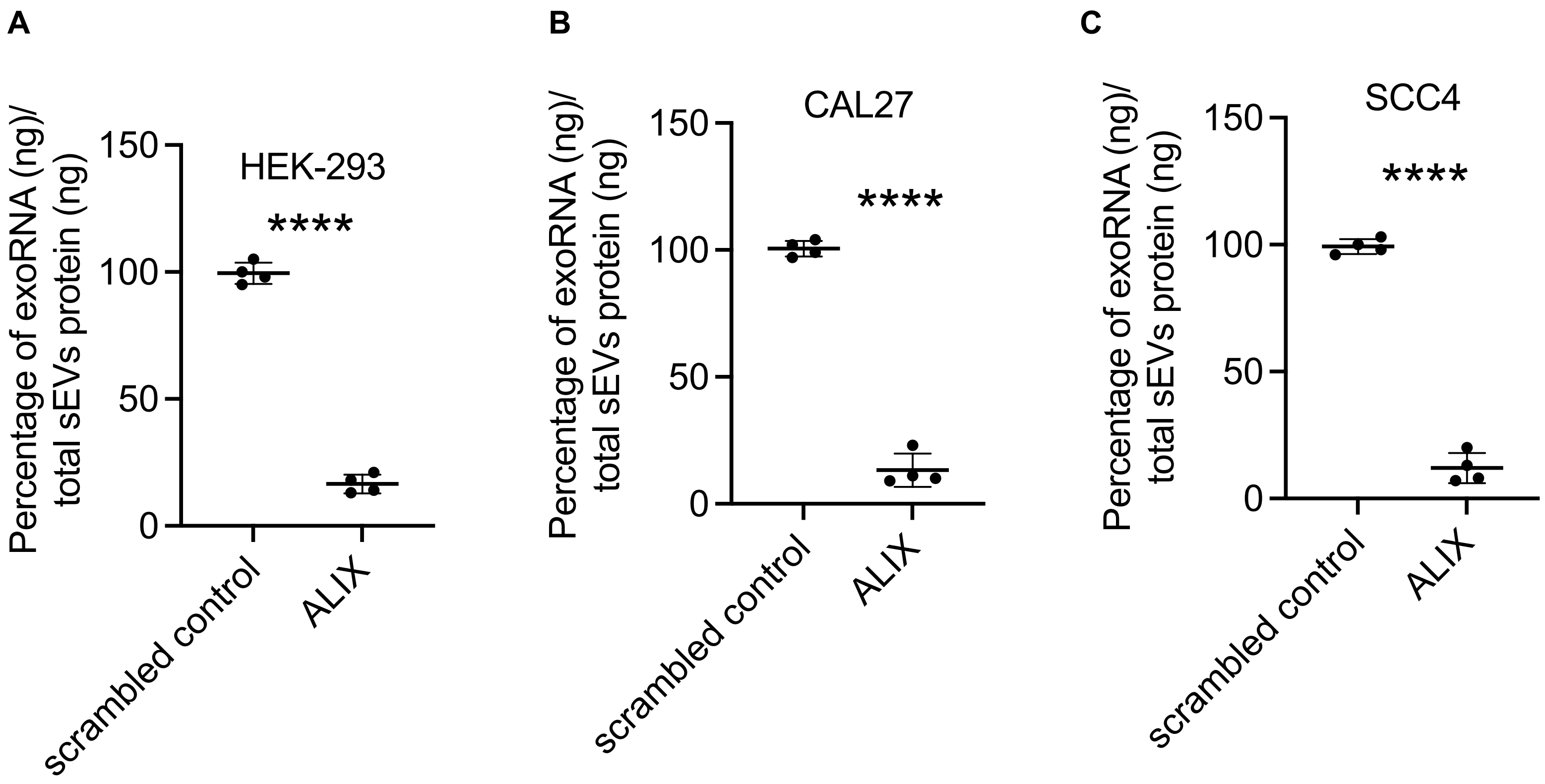

Figure S2- (a-c) Percentage change of total exo-RNA (ng) normalized based on the protein in sEVs, isolated from siRNA silenced ALIX HEK-293, CAL27 and SCC4 cells. Dot plots represent a mean ± standard deviation (SD). Student's T test was used for pairwise comparison. n = 4 per group. ****$p < 0.0001$.

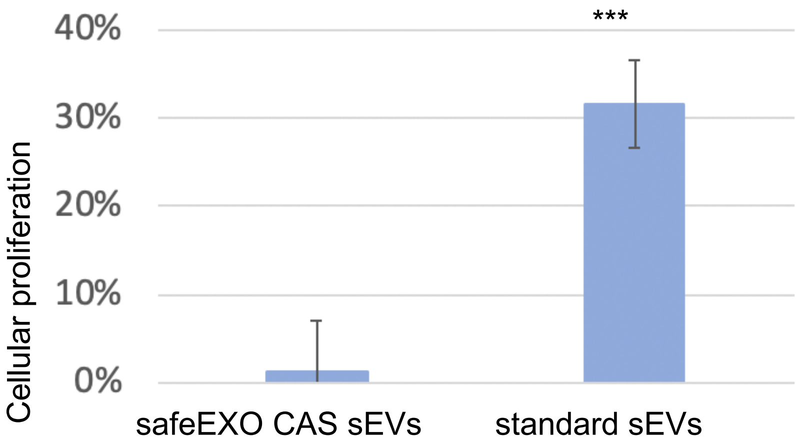

Figure S3- Bar plot showing the change in cellular proliferation following 72 hours of control administration of 25ug/ml safeEXO-CAS (derived from CAL27 after silencing Alix) and standard CAL27 sEVs. Bar graph represents mean ± standard deviation (SD). Student's T test was used for pairwise comparison. n = 4 per group. ***$p < 0.001$.

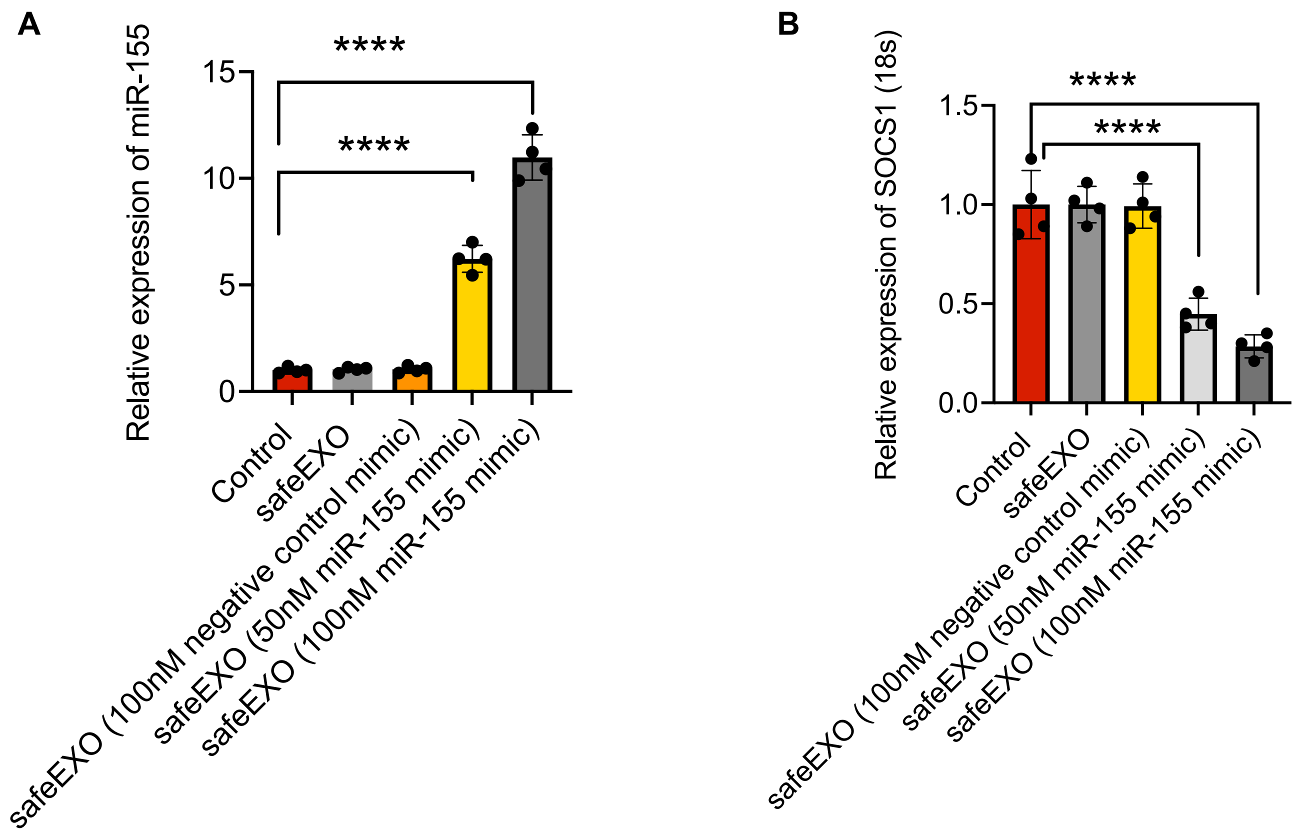

Figure S4- (a&b) Cells were seeded 1 day before treatment and different treatment conditions and controls were applied for 24 hours. Relative expression of miR-155 and miR-155 direct target (SOCS1) expression were measured by q-RTPCR. SOCS1 mRNA levels were significantly increased after exosome-mediated inhibition of miRNA-155. Cel-39 was used for normalizing miR-155. 18s was used as an internal normalizer for SOCS1. The data represents 4 independent experiments and presented as mean ± standard deviation (SD). One-way ANOVA was use for comparison of multiple group and Student's T test was used for pairwise comparison **** $p<0.0001$

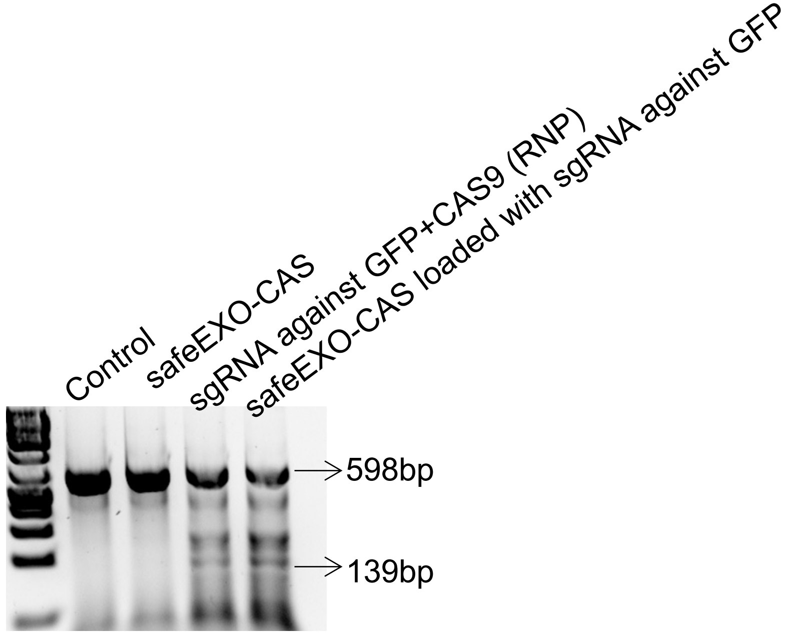

Figure S5- SafeEXO-CAS-mediated non-homologous end joining (NHEJ) genome editing. T7 endonuclease assay against the GFP in HEK-293 cells. Cells were treated with different safeEXO or safeEXO-CAS.

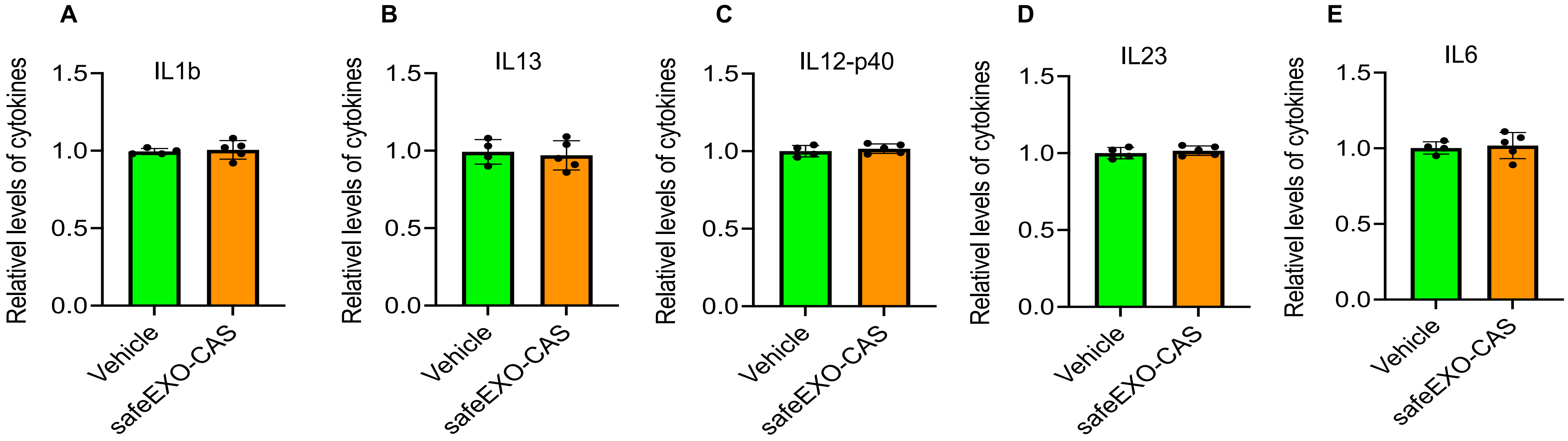

Figure S6- (a-e) The level of cytokines was identified in the mouse plasma using a LEGENDplex assay (BioLegend). The cytokine levels were normalized and presented based on the mean of the vehicle group. Data presented as mean ± standard deviation (SD). Student's T-test was used for pairwise comparison. n = 4-5 per group.

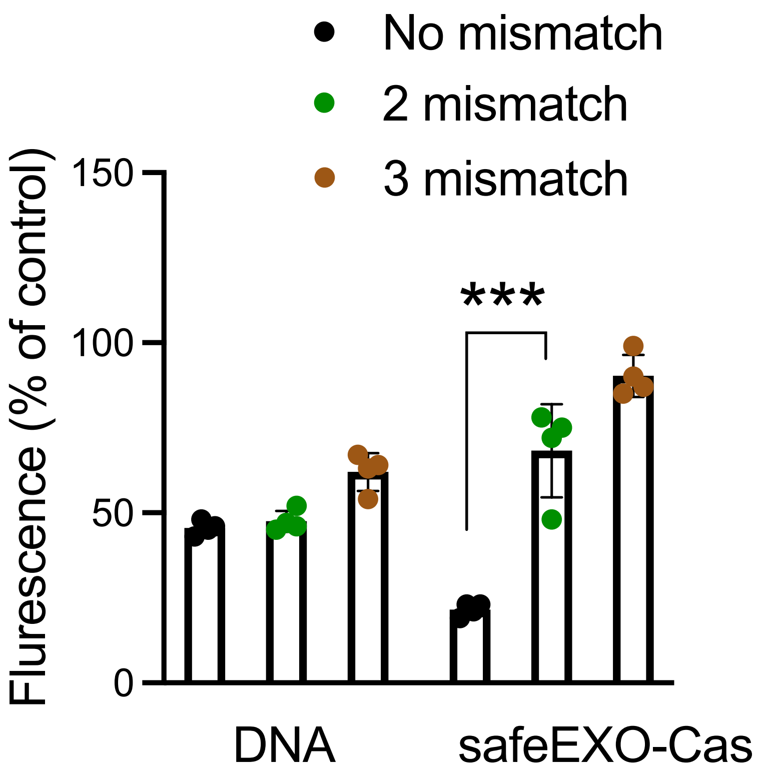

Figure S7- The off-target effect was identified in GFP reporter cell lines and gRNAs with 1-3 mismatch. Data presented as mean ± standard deviation (SD). Student's T test was used for pairwise comparison. n = 4 per group. ***p < 0.001.

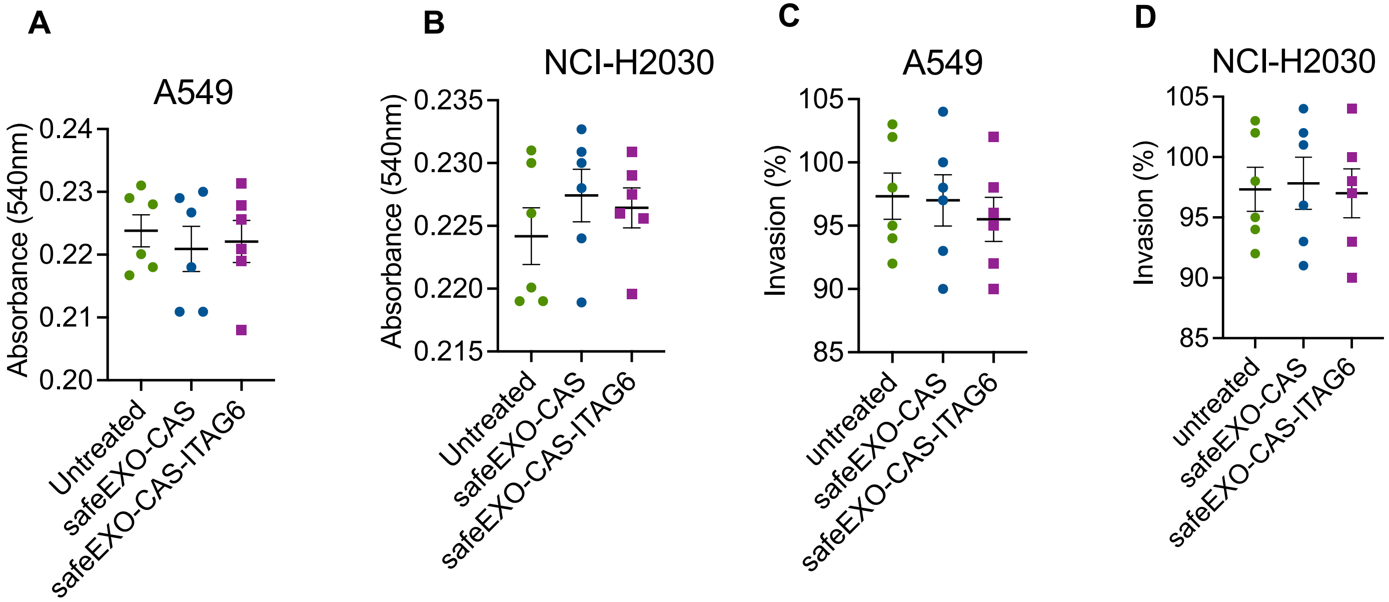

Figure S8- MTT assay (a,b) and transwell invasion assay (b,c). Detection of cell proliferation and cell migration 72h after incubation of lung cancer cell lines (A549 and NCI-H2030) with 25ug of safeEXO-CAS or safeEXO-CAS-ITGA6 in culture media. For the invasion assay, the percentage of cells was normalized based on the untreated condition. Data presented as mean ± standard error of the mean (SEM). The results are obtained from 2 independent experiments with 3 replicates each. Data were analyzed using one-way ANOVA.

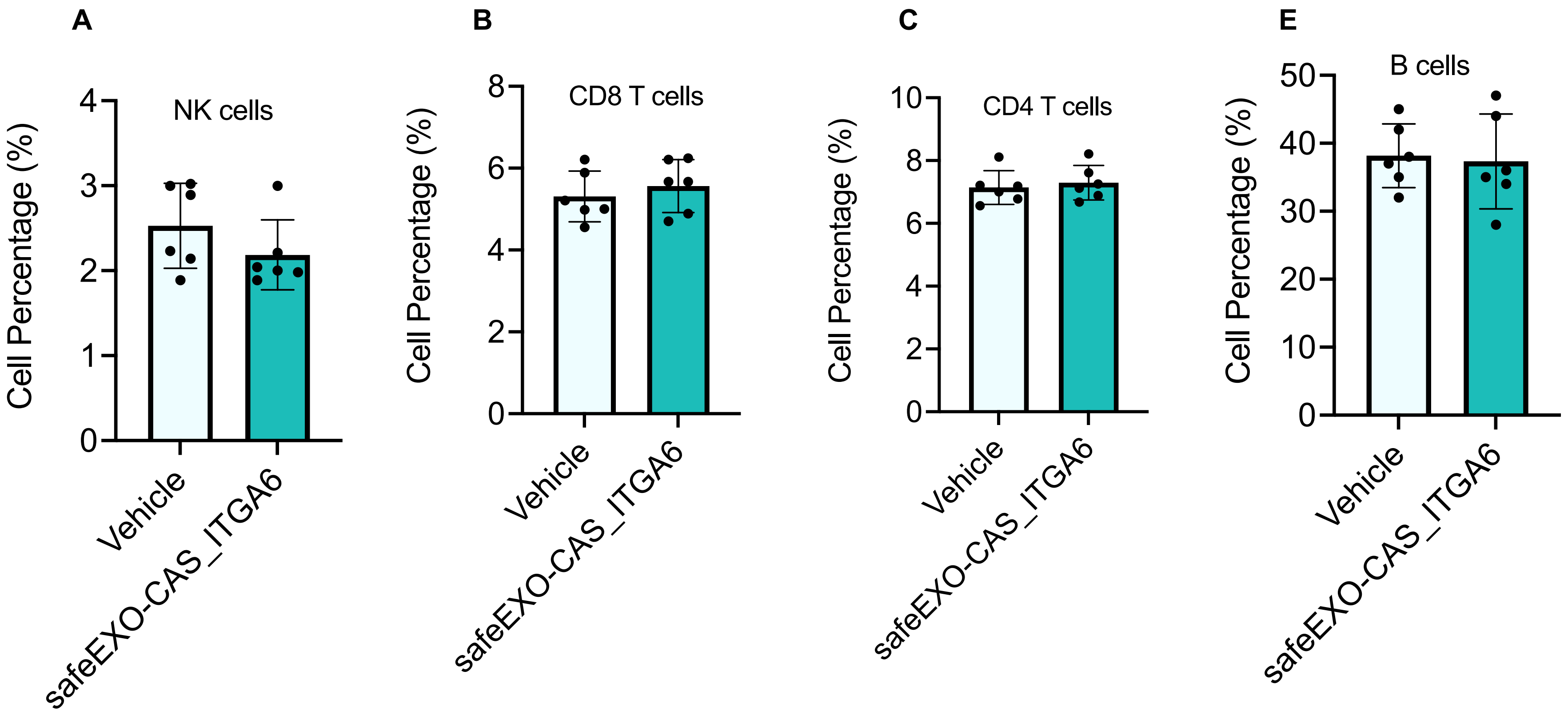

Figure S9- (a-e) Percentage of immune subtypes frequency in CD8^{+}T cells, B cells, monocytes, NK cells, neutrophils, and CD^{+}4 T cells in bone marrow (n=6 per group). Data are represented as mean ± standard deviation (SD).

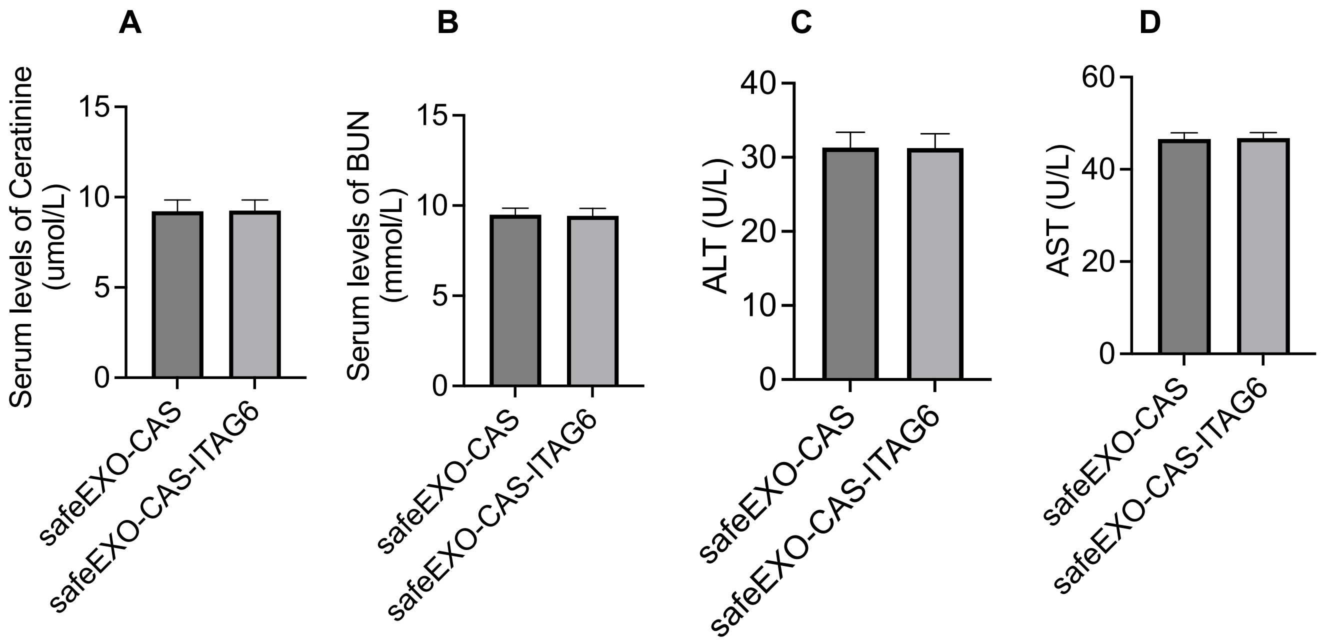

Figure S10- (a-d) Mice serum levels of Creatinine, blood urea nitrogen (BUN), ALT, and AST in safeEXO-CAS injected and safeEXO-CAS-ITGA6 treated mice. (N=4 per group)

Table S1- Mice toxicology panel (3 mice included in every comparison)

	Vehicle mice	Vehicle mice	Vehicle mice	safeEXO-CAS injected mice	safeEXO-CAS injected mice	safeEXO-CAS injected mice	P-value
**Phosphorus**	7.2	7.6	7.4	7.6	7.7	7.6	ns
**Uric Acid**	1.9	1.9	1.8	1.9	1.9	1.9	ns
**Calcium**	6	6.1	5.9	5.8	6	5.9	ns
**Glucose**	99	98	98	99	98	97	ns
**Total Protein**	3.2	3.2	3.1	3.1	3.2	3.2	ns
**Albumin**	1.6	1.6	1.7	1.7	1.7	1.7	ns
**ALP**	146	145	155	149	147	147	ns

ns: non-significant
